# NRF1 promotes primordial germ cell development, proliferation and survival

**DOI:** 10.1111/cpr.13533

**Published:** 2023-08-04

**Authors:** Pengxiang Wang, Jun Su, Junpeng Wang, Yilin Xie, Wei Chen, Jinhai Zhong, Yuan Wang

**Affiliations:** ^1^ Shanghai Key Laboratory of Regulatory Biology Institute of Biomedical Sciences and School of Life Sciences, East China Normal University Shanghai China; ^2^ Department of Animal Sciences, College of Agriculture and Natural Resources Michigan State University East Lansing Michigan USA

## Abstract

Primordial germ cells (PGCs) are the germline precursors that give rise to oocytes and sperm, ensuring the continuation of life. While the PGC specification is extensively studied, it remains elusive how the PGC population is sustained and expanded after they migrate to embryonic gonads before birth. This study demonstrates that NRF1, a known regulator for mitochondrial metabolism, plays critical roles in post‐migrating PGC development. We show that NRF1 protein level gradually increases in post‐migrating PGCs during embryonic development. Conditional *Nrf1* knockout from embryonic germ cells leads to impaired PGC proliferation and survival. In addition, NRF1 may also actively drive PGC derivation from pluripotent stem cells. Using whole genome transcriptome profiling and ChIP‐seq analyses, we further reveal that NRF1 directly regulates key signalling molecules in PGC formation, transcription factors in proliferation and cell cycle and enzymes in mitochondrial metabolism. Overall, our findings highlight an essential requirement of NRF1 in regulating a broad transcriptional network to support post‐migrating PGC development both in vitro and in vivo.

## Graphical abstract

During embryonic days 8.0–12.5, PGCs migrate to genital ridges, accompanied with concurrent genome‐wide DNA demethylation. NRF1 expression elevates during this developmental window, binds to unmethylated promoters, and activates transcription of genes involved in PGC specification, cell proliferation and enzymes in mitochondrial metabolism, which favours PGC development both in vivo and in vitro.

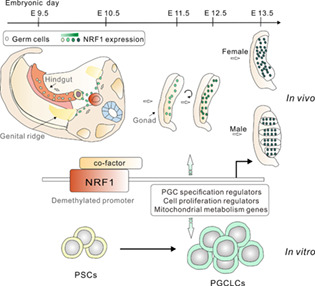

## 1 INTRODUCTION

Primordial germ cells (PGCs) are the very first germline precursors that give rise to oocytes and sperm, ensuring the continuation of life.^1^, ^2^, ^3^ In mice, PGC precursors emerge in proximal posterior epiblasts around embryonic day (E) 6.25 and establish a cluster of ~25 PGCs, segregating from surrounding somatic lineages at E7.25.^2^ Starting at E8.0, these nascent PGCs incorporate into the hind gut, migrate bilaterally along the dorsal mesentery, and populate the newly formed genital ridges at E10.5–12.5.^1^, ^2^, ^3^ During this period, PGCs proliferate steadily, increasing to ~1000 in number.^1^, ^2^, ^3^ After E12.5, when sex‐specific features of gonads appear, the fate of PGCs starts to diverge in male and female embryos. In the male genital ridges (embryonic testes), PGCs undergo transient proliferation (reaching ~25,000 cells in number) before arresting at mitotic G0/G1 phase as pro‐spermatogonia (also referred as gonocytes) around E14.5–16.5. Meanwhile, in embryonic ovaries, PGCs proliferate before E14.5 and become oogonia, which then enter meiosis and arrest at prophase of the first meiotic division as oocytes.^4^, ^5^, ^6^ Sex determination marks the end of the primordial phase of germ line development and the beginning of spermatogenesis or oogenesis. Any defects in PGC formation, migration and proliferation can result in both female and male infertility.

Transcription factors (e.g., PRDM1/BLIMP1, PRDM14 and TFAP2C) and signalling molecules (e.g., BMPs and WNT) play crucial roles in PGC specification.^3^, ^7^, ^8^, ^9^, ^10^ In addition, epigenetic reprogramming occurs during PGC development with extensive genome‐wide DNA demethylation and erasure of sex‐specific imprints.^11^, ^12^, ^13^ In mice, PGC precursors at ~E6.75 initially have a genome‐wide methylation level that is indistinguishable from their somatic neighbours.^14^ Passive DNA demethylation begins almost concurrently with PGC specification, migration and colonisation into the gonads through downregulation of DNMT3a/b.^11^, ^14^, ^15^, ^16^, ^17^, ^18^, ^19^ Then, the DNA demethylation continues at the remaining imprinting regions of the PGC genome, facilitated by upregulated TET methylcytosine dioxygenases.^15^ By E13.5, the majority of the genome in post‐migration PGCs is hypomethylated.^11^, ^14^, ^15^, ^16^, ^17^, ^18^, ^19^ Another key feature of proliferating PGCs is their higher mitochondrial metabolism, compared to their surrounding somatic cells. Inhibiting glycolysis favours the formation of PGC‐like cells (PGCLCs) from pluripotent stem cells (PSCs), while repressing mitochondrial respiration reduces PSC differentiation into PGCLCs.^20^ However, it is yet to be elucidated how mitochondrial metabolism is upregulated to support PGC proliferation before sex‐determination.

Nuclear respiratory factor 1 (NRF1) is a transcription factor known for its role in mitochondrial biogenesis.^21^ NRF1 was first identified through its binding to the promoter of cytochrome C,^22^ and was subsequently shown to regulate the expression of genes involved in the mitochondrial respiratory chain.^23^, ^24^ We recently reported that during spermatogenesis, NRF1 bound to hypomethylated CpG‐rich promoter regions of germ cell‐specific genes and directly activated their transcriptions.^25^ In the current study, we found a higher NRF1 expression in post‐migrating PGCs during the embryonic stages, compared to their surrounding somatic cells, likely due to the gradually hypomethylated PGC genome. In addition, NRF1 was required for PGC proliferation and survival both in vitro and in vivo, and it actively promoted PGC derivation from PSCs when ectopically expressed. Through RNA‐seq and ChIP‐seq analyses, we further revealed that NRF1 regulated a broader transcriptional network crucial for maintaining mammalian PGC development.

## 2 EXPERIMENTAL MATERIALS AND METHODS

### 2.1 Animal models

The *Nrf1* conditional knockout mice were generated in a previously published study.^25^ *Tnap‐Cre* mice were previously reported^26^ and a kind gift from Dr. Guoliang Xu at the Chinese Academy of Science. Both mouse strains were maintained under the C57BL/6 background. To get *Nrf1* ^*−/f*^ *‐Cre* mice, *Nrf1* ^*f/f*^ female mice were crossed with *Nrf1* ^*+/f*^ *‐Cre* male mice. All animal experimental procedures were approved by the Institutional Animal Care and Use Committee at East China Normal University and conducted in compliance with the regulatory guidelines.

### 2.2 Histology study and histoimmunofluorescence (IHF) assays

For histology analyses, tissues were fixed in 4% paraformaldehyde (PFA), followed by dehydration, embedded in paraffin and processed into 5 μm sections for staining with haematoxylin (Sigma‐Aldrich, MHS16) and eosin (Sigma‐Aldrich, HT110216). Images were obtained with a Leica microscope (Leica, DM4000B). IHF was performed as previously described by Zhang et al.^27^ Briefly, embryonic gonads were fixed with 4% PFA in PBS at 4°C for 2 h and then embedded in paraffin. The 5 μm‐thick gonad sections were incubated with primary antibodies followed by washing three times with PBS containing 0.1% Triton X‐100. Nuclei were stained with DAPI after blotting with fluorochrome‐conjugated second antibodies. Images were obtained with a confocal microscope (Andor‐Oxford Instruments, DF505). Primary antibodies used in this study: OCT4 (used as 1:100 dilution, Santa Cruz, sc‐5279), STELLA (Sigma, 1:300, MAB4388); NRF1 (1:400, Abcam, ab175932), DDX4 (1:400, Abcam, ab27591), Ki67 (1:400, Abcam, ab15580) and cleaved Caspase‐3 (1:400, Cell Signalling Technology, #9661).

### 2.3 Plasmid construction for NRF1 overexpression (OE)

We used the CRISPR/dCAS9 gene editing system to activate endogenous *Nrf1* expression according to the published protocol.^28^ Briefly, sgRNA targeting *Nrf1* was cloned into Lenti‐sgRNA (MS2)_puro (Addgene, #73795). The dCas9‐VP64 (Addgene, #61425) and MS2‐P65‐HSF1 (Addgene, #61426) were introduced simultaneously into PSCs to activate endogenous *Nrf1* expression. To construct NRF1 OE plasmid, *Nrf1* cDNA was amplified from mouse PSCs and cloned into *SalI* and *NheI* sites of a modified pInducer20 (*p20*) vector (Addgene, #44012). The correctly inserted sgRNA sequence and *Nrf1* cDNA in the expression vectors were confirmed by Sanger sequencing. The sequences of sgRNA for Nrf1 activation and primers for *Nrf1* cDNA cloning are listed in Table S4.

### 2.4 Cell culture, transfection and viral infection

293T cells were cultured in DMEM (Gibco) with 10% fetal bovine serum (FBS, Gibco) and transfected with plasmids using polyethyleneimine (Sigma) for viral packaging. PSCs were cultured in DMEM (Gibco) with 15% FBS (Gibco), 1 μM PD0325901(Stemgent), 3 μM CHIR99021 (Stemgent), 1000 units/mL LIF (Gibco), 0.05 mM *β*‐mercaptoethanol, 2 mM l‐Glu, 0.1 mM NEAA, 100 units/mL penicillin and 0.1 mg/mL streptomycin on feeder‐free wells coated with 0.2% gelatin. For viral infection of PSCs, lentivirus was packaged in 293T cells. Dissociated PSCs were mixed with lentivirus with MOI at 10 and 5 μg/mL polybrene. Cell medium was changed at 12 h post‐infection. Cells were passed every 2–3 days; 400–800 μg/mL G418 or 1 μg/mL puromycin was used to select for transgene or sgRNA expressing cells.

### 2.5 PGCLC derivation from PSCs

The embryoid bodies (EBs) were generated from 1.5 × 10e6 PSCs in 10 cm petri dishes with differentiation medium including IMDM with 15% FBS, 0.1 mM NEAA, 1 mM sodium pyruvate, 2 mM l‐Glu, 0.1 mM α‐monoglycero, 100 units/mL penicillin, 0.1 mg/mL streptomycin and 50 μg/mL l‐ascorbic Acid. All medium components referenced above were purchased from Gibco. Cells were cultured on a shaker in the cell incubator, with the medium changed every 2 days. For *p20* vector‐based gene expression by doxycycline (DOX) induction, 1.5 μg/mL DOX were added during PSC differentiation from day 2 to 5.

### 2.6 Cell sorting and analysis by flow cytometry

The EBs were dissociated with TrypLE into single cells, washed with PBS/0.1% BSA, and collected by centrifugation. Cell clumps were removed by a 40 μm cell strainer (BD Biosciences). Dissociated cells were incubated with indicated antibodies for further analyses. For Ki67 staining, 3 mL of cold 70% ethanol drop by drop was added to the cell pellet with gentle vortex. Resuspended cells were kept at −20°C for 1 h, followed by washing three times with cell staining buffer (BioLegend, Cat. # 420201) and then resuspended by cell staining buffer into ~5 × 10^5^/mL; 100 μL cell suspension was incubated with Ki‐67 antibody at room temperature in the dark for 30 min, and then washed twice with cell staining buffer before being analysed by flow cytometry. For Annexin V, staining was performed according to the manufacturer's protocol, co‐stained with 5 μg/mL propidium iodine (PI). Cells were analysed on Fortessa or BD LSR II, sorted with ARIA II or BD Influx flow cytometers (all from BD Biosciences). All antibodies used in flow cytometry were purchased from BioLegend: APC‐SSEA1 (Cat. # 125618), BV421‐Ki67 (Cat. # 652411) and BV421‐Annexin V (Cat. # 640923).

### 2.7 RNA‐Seq and bioinformatic analyses

For RNA‐seq, total RNAs from sorted BV+ PGCLCs at day 5 of BVSC PSC differentiation were extracted with RNAiso plus (Takara, Cat. # 9109). Biological duplicates or replicates were prepared as indicated. Multiplexed libraries with different indexes were constructed and sequenced on an Illumina NovaSeq instrument using 2 × 150 bp paired‐end configuration. After removing adaptor sequences, trimmed reads with length shorter than 75 bp, and low‐quality sequences/reads by FastQC (v0.10.1) and Cutadapt V1.9.1, clean sequences were mapped to the mouse genome (Mus_musculus_GRCm39.107) using Hisat2 (v2.0.1). Read counts were obtained with HTSeq (v0.6.1) to get gene expression as FPKM (expected number of fragments per kilobase of transcript sequence per million base pairs sequenced). DESeq2 (v1.26.0) and TMM were used to normalised read counts and obtain the differential gene expression across groups. Goseq (v1.34.1) was used for Gene Ontology terms that annotate enriched genes with the Log2 (Fold Change) cutoff as 0.58 and the *p* value <0.05. Raw RNA‐seq data were uploaded to the GEO repository and accessible to the public (GSE231662).

### 2.8 ChIP‐Seq and bioinformatic analyses

For ChIP‐seq analyses, we performed ChIP on 8 × 10‐e6 sorted BV+ PGCLCs using SimpleChIP® Plus Enzymatic Chromatin IP Kit (Cell Signalling Technology, #9005) with a NRF1 antibody (Abcam, Ab175932). Immunoprecipitated DNA was quantified using a Qubit 3.0 Fluorometer (Invitrogen) and qualified with an Agilent Bioanalyzer 2100 (Agilent Technologies). Biological triplicates were prepared. DNA libraries were constructed and sequenced with an Illumina NovaSeq instrument (Illumina) using a 2 × 150 paired‐end configuration. After removing adaptor sequences, sequences with Phred value <20, and trimmed reads with length shorter than 75 bp by Cutadapt V1.9.1, clean sequences (Q20 > 97.5% in all samples) were aligned to mouse genome (Mus_musculus_GRCm39.107) using the software Bowtie2 (version 2.2.6). Peak quality, peak calling and peak annotation were used MACS2 and ChIPseeker software. The peaks of different experimental groups were combined with bedtools software (version, 2.25.0), and differential analysis was performed using edgeR (V3.24.1) from the bioconductor software package. Peaks at the specific genomic regions were obtained with Integrative Genomic Viewer. The peaks that appeared in biological duplicates or triplicates were further analysed for their gene functional categories using GOSeq (v1.34.1). Raw ChIP‐seq data were uploaded to the GEO repository and accessible to the public (GSE231661).

### 2.9 RT‐PCR, PCR and real‐time PCR

Total RNAs were extracted with RNAiso plus and reverse‐transcribed using a PrimeScrip RT reagent Kit (TAKARA, RR037A) according to the manufacturers' instructions. PCR and real‐time PCR were performed as described previously by Zhang et al.^29^ Primers used in this study were listed in Table S4.

### 2.10 Statistical analysis

Data were presented as mean ± SEM. All experiments were performed independently three times or more unless otherwise stated. Statistical comparisons of between‐group means were performed using unpaired Student's *t*‐test and the Prism Graphic software.

## 3 RESULTS

### 3.1 Conditional *Nrf1* knockout in germ cells impairs the development of post‐migration PGCs

**FIGURE 1:**
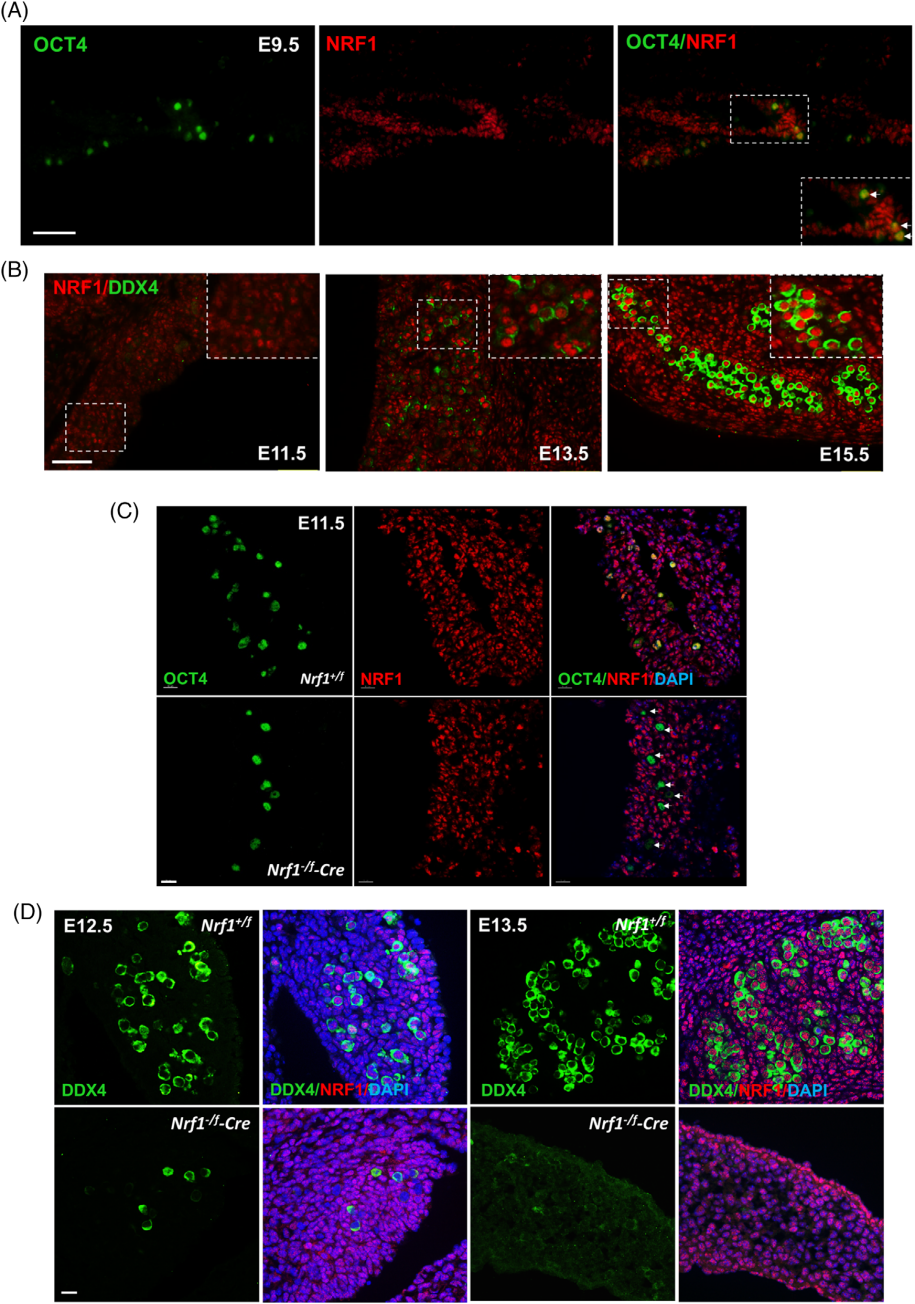
NRF1 is required for primordial germ cells (PGC) development. (A) NRF1 expression in PGCs was detected by immunohistofluorescent (IHF) on E9.5 mouse embryos with NRF1 and OCT4 antibodies. (B) IHF assays were performed on E11.5–15.5 embryonic gonads with antibodies against NRF1 and germ cell‐specific protein DDX4. (A,B) Scale bar: 50 μm. Inserts showed blow‐up images of representative regions. (C,D) IHF assays were performed on E11.5–13.5 gonads from wildtype control embryos and *Nrf1* conditional knockout embryos by *Tnap‐Cre* in PGCs (*Nrf1* ^*−/f*^ *‐Cre*), with antibodies against NRF1 and OCT4 (C), or NRF1 and DDX4 (D), counterstained with a nucleus dye, DAPI. Scale bar: 15 μm. (C) White arrows indicate OCT4+ PGCs with NRF1 expression completely abolished.

To determine whether NRF1 participates in PGC development, we first assessed its expression levels in the migrating and post‐migrating PGCs during embryonic development using immunohistofluorescent (IHF) assays. We found that NRF1 expression was relatively low in OCT4+ or STELLA+ PGCs at E9.5 and E11.5 but gradually elevated during embryonic development (Figure 1A,B and Figure S1A). In addition, NRF1 proteins expressed at a higher level in PGCs compared to that of surrounding somatic cells at E13.5 and E15.5 (Figure 1B), suggesting a potential requirement of NRF1 in post‐migrating PGCs.

Because NRF1 null mutation leads to embryonic lethality between E3.5 and 6.5,^30^ to understand the role of NRF1 in PGCs at the later stages, we generated PGC‐specific *Nrf1* knockout mice with a *Tnap‐Cre* driver, in which CRE started to express in PGCs at E9.5^26^ and deleted the exon 4 from *Nrf1* gene locus (Figure S1B,C). We found that the number of OCT4+ post‐migrating PGCs started to reduce between E11.5 and E12.5 upon conditional *Nrf1* knockout (*Nrf1* ^*−/f*^ *‐Cre*, Figure 1C,D and Figure S2A). At E13.5, few DDX4+ germ cells remained intact in *Nrf1* deleted gonads (Figure 1D and Figure S2A). Taken together, these data suggest that a high NRF1 expression level is required for maintaining post‐migrating PGC development in mice.

### 3.2 *Nrf1* deletion from PGCs leads to infertility of both male and female mice

**FIGURE 2:**
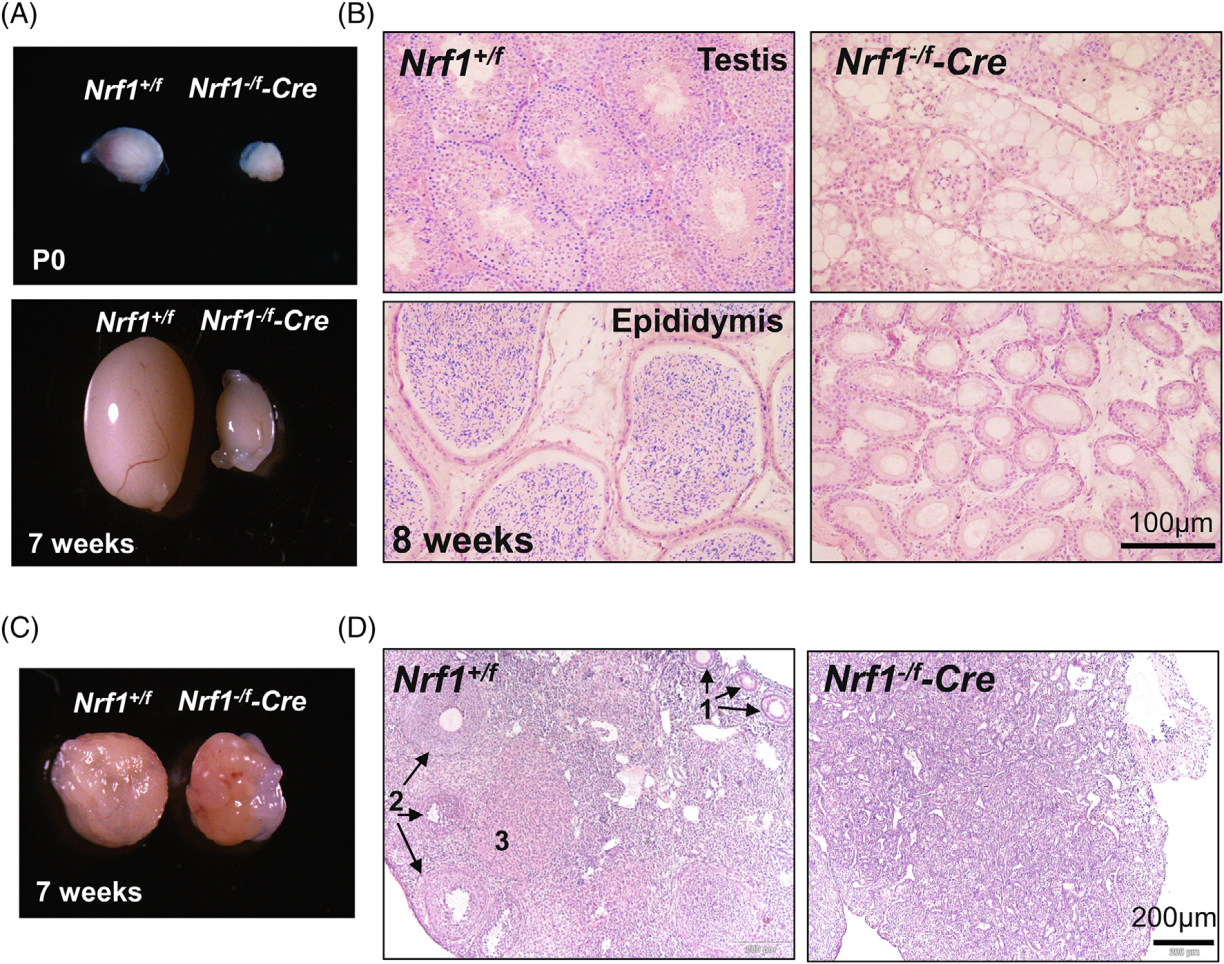
*Nrf1* knockout leads to infertility in both male and female mice. (A) Morphology of testes from *Nrf1* ^*−/f*^ *‐Cre* mice and their wildtype littermate controls at P0 and 7 weeks old. (B) Histology on testes and epididymis from *Nrf1* ^*−/f*^ *‐Cre* mice and their wildtype littermate controls at 8 weeks old. (C) Morphology of ovaries from *Nrf1* ^*−/f*^ *‐Cre* mice and their wildtype littermate controls at 7 weeks old. (D) Histological study on ovary sections from *Nrf1* ^*−/f*^ *‐Cre* mice and their wildtype littermate controls at 7 weeks old. Black arrows and numbers indicate follicles at different developmental stages. 1: primary follicles; 2: secondary follicles; 3: corpus luteum.

To further prove NRF1 is essential for PGC development, we examined germ cell formation in both female and male embryos at E15.5. As expected, no DDX4+ embryonic germ cells were detected in either male or female *Nrf1* knockout gonads (Figure S2B,C). Consistent with these findings, we found both male and female *Nrf1* conditional knockout mice were infertile. The testes of *Nrf1* ^*−/f*^ *‐Cre* male mice were visibly smaller at postnatal day (P) 0, which were significantly reduced at 7 weeks old (Figure 2A,B). Histology and IHF showed that no DDX4+ germ cells were detected at *Nrf1* ^*−/f*^ *‐Cre* neonatal testes (Figure S3A). Consequently, Sertoli‐only phenotype was observed in adult testes upon *Nrf1* deletion by *Tnap‐Cre* (Figure 2A,B). No sperm was detected in the epididymis of these mice either (Figure 2B). In female mice, compared to wildtype ovaries containing follicles at various developmental stages, *Nrf1* ^*−/f*^ *‐Cre* ovaries were slightly smaller at 7 weeks old (Figure 2C). Ovaries at either P7 before puberty (Figure S3B) or during adulthood (Figure 2C,D) contained no visible developing follicles or mature oocytes upon *Nrf1* deletion. In summary, these data confirm that NRF1 plays an essential role for embryonic germ cell development in both male and female animals.

### 3.3 NRF1 deficiency compromises the survival and proliferation of post‐migration PGCs

**FIGURE 3:**
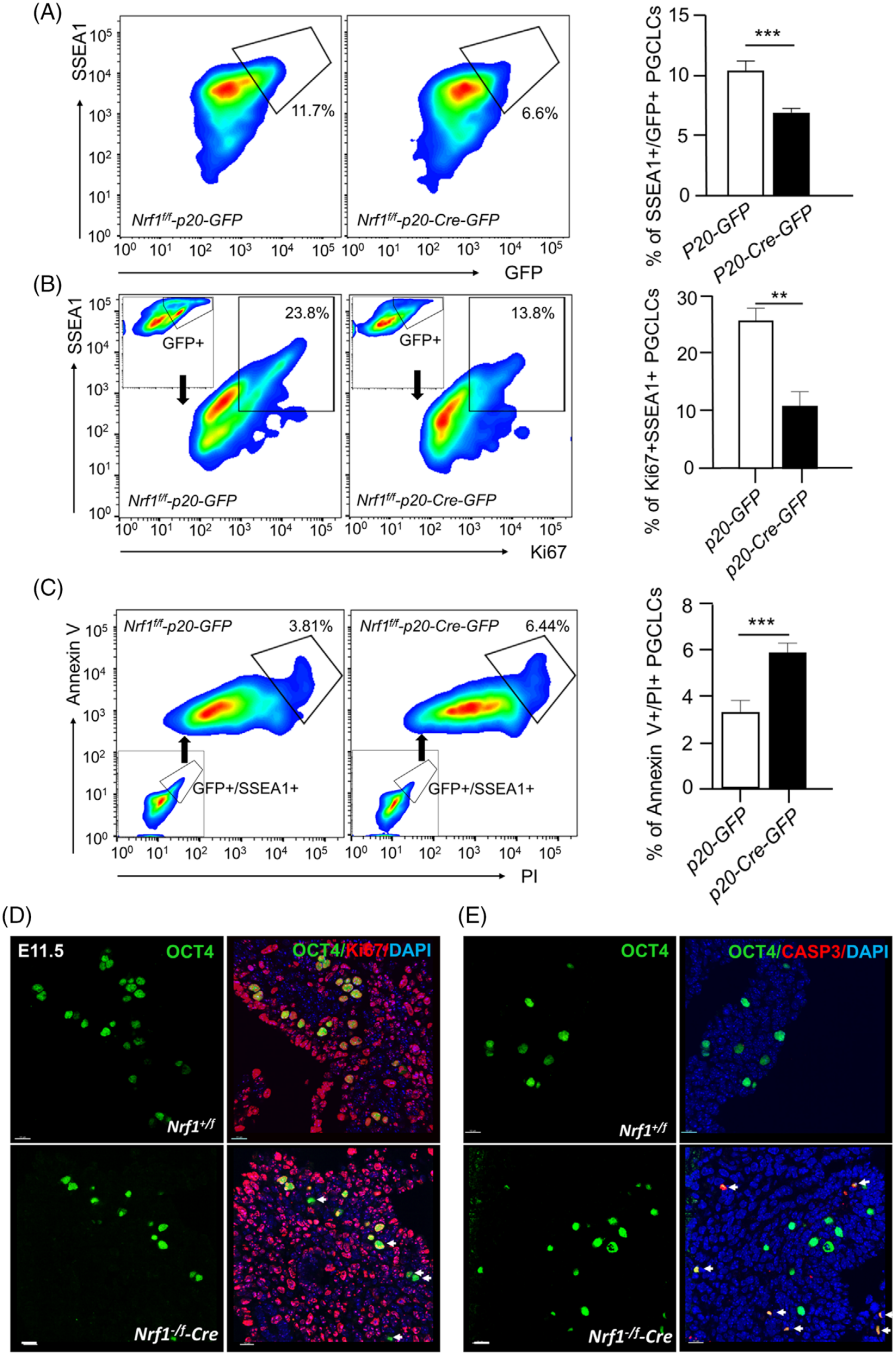
NRF1 is required for the proliferation and survival of postmigrating primordial germ cells (PGCs). (A) Flow cytometry analyses on differentiated *Nrf1* ^*f/f*^ PSCs introduced with *p20‐GFP* or *p20‐Cre‐p2a‐GFP*. GFP+ PGC‐like cells (PGCLCs) were detected by SSEA1 staining. The percentages of GFP+/SSEA1+ PGCLCs were shown on the right. (B) GFP+/SSEA1+ PGCLCs from (A) were analysed by flow cytometry using a BV421‐conjugated antibody against Ki67. (C) GFP+/SSEA1+ PGCLCs from (A) were co‐stained with BV421‐conjugated Annexin V and propidium iodide (PI), and samples were then analysed by flow cytometry. (A–C) Data were presented as mean ± SEM from three independent experiments. ***: *p* < 0.001. (D,E) Immunohistofluorescent (IHF) on genital ridges from *Nrf1* ^*−/f*^ *‐Cre* embryos and their wildtype littermates at E11.5 with antibodies against OCT4 and Ki67 (D), or OCT4 and cleaved Caspase 3 (CASP3) (E). White arrows indicate OCT4+/Ki67 non‐proliferating PGCs (D) or OCT4+/Caspase 3+ apoptotic PGCs (E) in *Nrf1* ^*−/f*^ *‐Cre* embryos. Scale bars: 10 μm.

The in vitro differentiation of PSCs generates a large quantity of PGCLCs, and thereby greatly facilitating the molecular studies of germ cell specification. We and others have previously shown that SSEA1+ cells in differentiated PSCs represent post‐migrating PGCs developed in vivo.^31^, ^32^, ^33^ To explore the underlying functional mechanisms by which NRF1 regulates PGC development, we derived *Nrf1* ^*f/f*^ PSCs and examined the formation of SSEA1+ PGCLC upon *Nrf1* deletion. We introduced a DOX‐inducible CRE‐p2a‐GFP‐expressing vector into these *Nrf1* ^*f/f*^ PSCs (Figure S1C). An empty vector conditionally expressing GFP was used as a control to exclude potential side effects due to DOX treatment. In GFP+ cells with *Nrf1* knockout induced by DOX treatment, we found that SSEA1+ PGCLCs in differentiated PSCs were significantly reduced (Figure 3A). Interestingly, we further found that the proliferating PGCLCs labelled by Ki‐67+, a proliferation marker,^34^ were also dramatically decreased upon *Nrf1* deletion (Figure 3B). By contrast, a higher fraction of Annexin V+/PI+ apoptotic cells was detected in *Nrf1* knockout PGCLCs (Figure 3C).

We further confirmed these data in vivo using *Nrf1* ^*−/f*^ *‐Cre* embryos. We found that while Ki67 was readily detectable in post‐migrating PGCs from wildtype embryos at E11.5, few remaining PGCs were co‐stained positive for Ki67 and OCT4 in the gonads of *Nrf1* conditional knockout embryos (Figure 3D and Figure S3C). In addition, the signal of cleaved Caspase 3, an indicator of apoptotic cells,^35^ increased upon *Nrf1* deletion (Figure 3E and Figure S3D), suggesting that NRF1 is required for supporting the proliferation and survival of post‐migrating PGCs both in vitro and in vivo.

### 3.4 Enforced NRF1 expression promotes PGCLC formation from PSCs

**FIGURE 4:**
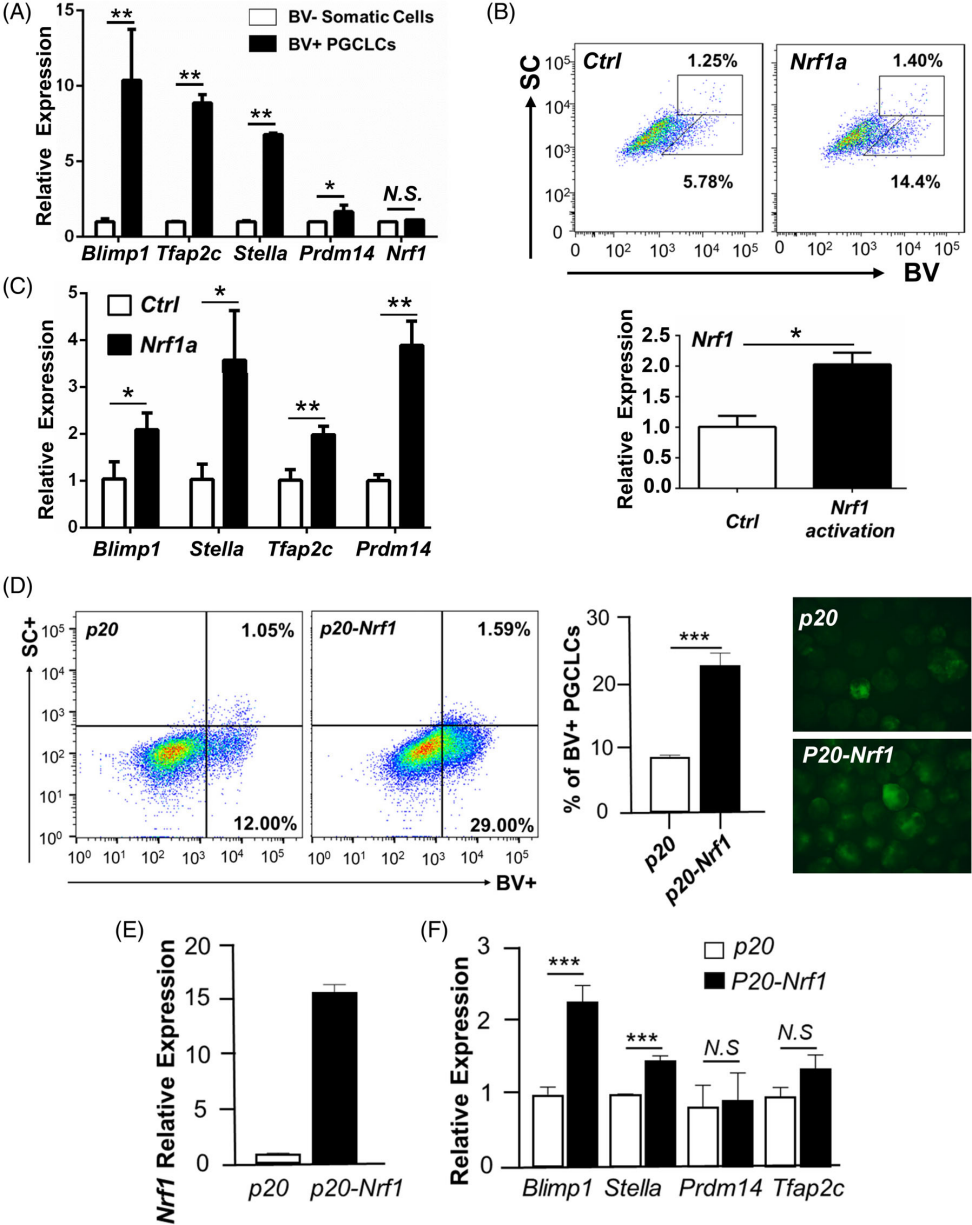
NRF1 promotes PGC‐like cell (PGCLC) formation from PSCs. (A) Relative mRNA levels of *Nrf1* and primordial germ cells (PGC) marker genes were measured by real‐time RT‐PCR in BV+ PGCLCs, compared to BV− somatic cells differentiated from BVSC PSCs. (B) BV+ PGCLCs were measured by flow cytometry upon endogenous *Nrf1* activation (*Nrf1a*), compared to an empty vector control (*Ctrl*). (C) Relative mRNA levels were measured by real‐time RT‐PCR on whole day 6 EBs upon endogenous *Nrf1* activation. (A–C) Data were presented as the mean ± SEM from two independent biological replicates. (D) Flow cytometry analyses of BVSC PSCs on day 5 of differentiation. NRF1 expression (*p20‐Nrf1*) was induced by DOX treatment from day 2 to 5 during PSC differentiation. An empty vector with the same DOX treatment was used as a control (*p20*). Representative results of flow cytometry were shown on the left, and the percentages of BV+ PGCLCs were shown in the middle panel. Right panel: representative images of EBs from BVSC PSCs on day 5 of differentiation. (E) The mRNA levels of *Nrf1* and PGC marker genes were measured by real‐time RT‐PCR on day 5 differentiated EBs. NRF1 expression was induced by DOX treatment from day 2 to 5 during PSC differentiation. (D–F) Data were presented as mean ± SEM from three independent experiments. (A–F) *: *p* < 0.05; **: *p* < 0.01. ***: *p* < 0.001. *N.S*.: no significance.

Published studies demonstrated that increased mitochondrial metabolism favours PGCLC formation from PGCs. Because NRF1 is known as a master regulator of mitochondrial biogenesis, we explored if enforced NRF1 promoted PGCLC specification from PSCs. We utilised a PSC reporter line expressing fluorescent proteins driven by PGC‐specific promoters (i.e., BVSC: Blimp1‐mVenus; Stella‐CFP).^36^ We isolated the BV+ PGCLCs from differentiated PSCs and measured that *Nrf1* expression by real‐time RT‐PCR. Consistent with relatively low expression of NRF1 in nascent^8^, ^36^ or migrating (Figure 1A and Figure S1A) PGCs in vivo, we observed no significant difference in *Nrf1* expression between BV+ PGCLCs and BV− surrounding somatic cells (Figure 4A).

Interestingly, when the endogenous NRF1 expression was activated by CRISPR/dCAS9‐VP64‐p65^28^ with sgRNAs targeting *Nrf1*, the formation of BV+ PGCLC from differentiated PSCs was elevated (Figure 4B), accompanied by upregulated expression of PGC marker genes including *Blimp1*, *Stella*, *Prdm14* and *Tfap2c* (Figure 4C).^36^ To exclude possible off‐target effects by CRISPR/dCAS9 system, we further confirmed this result by enforcing ectopic NRF1 expression with an *Nrf1* cDNA. We found that BV+ PGCLCs were increased upon continuous NRF1 OE in PSCs (Figure S4A). We observed the same phenomenon when exogenous NRF1 expression was induced by DOX treatment from day 2–6 of PSC differentiation (Figure 4D). Consistently, PGC regulators such as *Blimp1* and *Stella* were upregulated upon NRF1 enforced expression in differentiated PSCs (Figure 4E,F and Figure S4B). Taken together, our data suggest that NRF1 is not only a permissive player in germ cell development, but also an active driver to promote PGC specification from pluripotent cells.

### 3.5 NRF1 directly regulates genes critical for PGC formation, mitochondrial metabolism and cell proliferation

**FIGURE 5:**
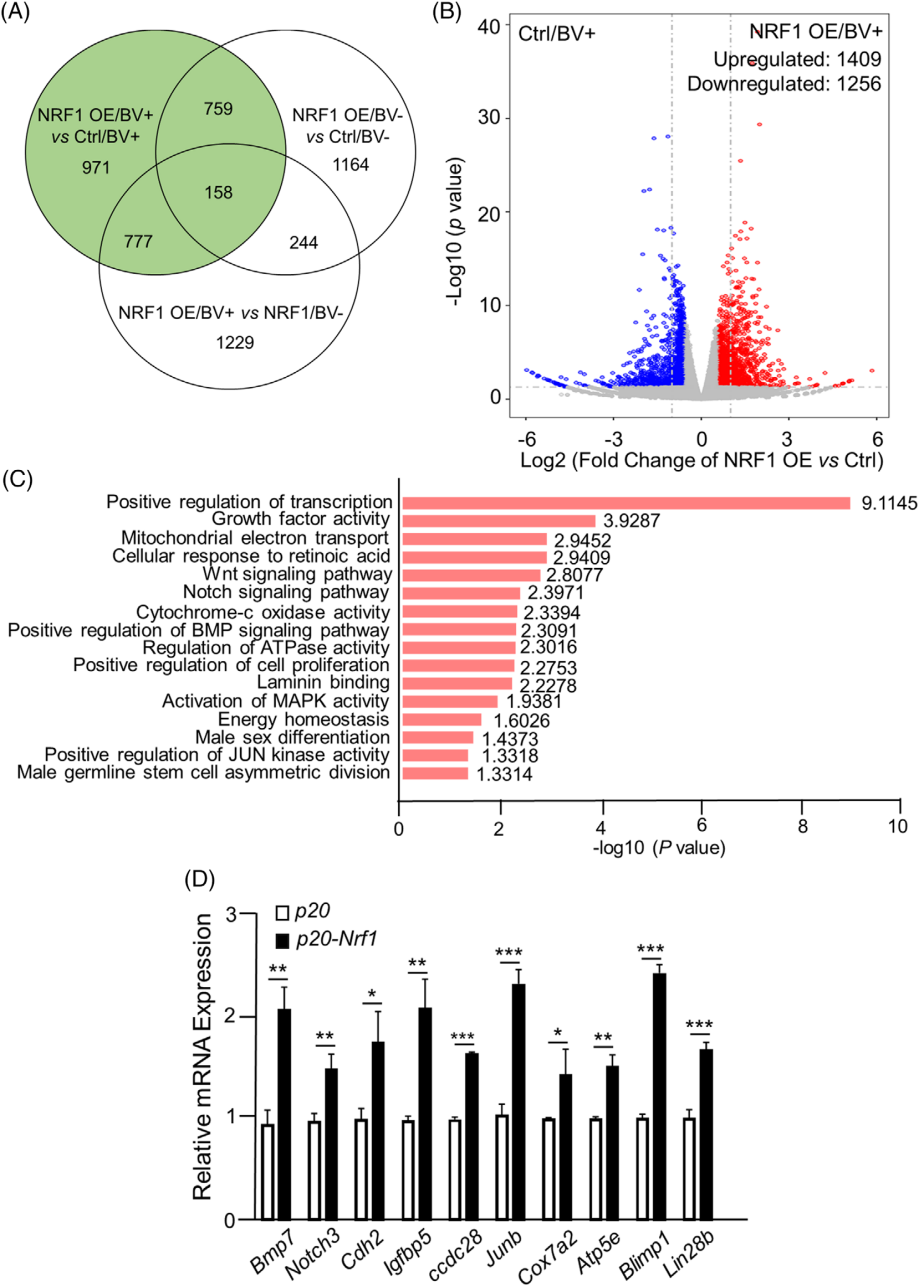
NRF1 regulates the transcription network that induces PGC‐like cell (PGCLC) derivation from PSCs. (A) Venn graph of differentially expressed genes from RNA‐seq analyses with a Log_2_ fold change >0.58 and a *p* value <0.05. (B) Volcano graph of genes detected by RNA‐seq analyses on BV+ PGCLCs with or without NRF1 overexpression (OE). (C) Gene ontology analyses of differentially expressed genes (Log_2_ fold change >0.58, *p* value <0.05) on BV+ PGCLCs with or without NRF1 OE. (D) Real‐time RT‐PCR analyses on BV+ PGCLCs with DOX‐induced NRF1 OE (*p20‐Nrf1*) or empty vector control (*p20*). Data were presented as the mean ± SEM from three independent biological replicates. *: *p* < 0.05; **: *p* < 0.01. ***: *p* < 0.001.

To understand the molecular mechanisms by which NRF1 actively drives PGC development from PSCs, we performed RNA‐seq analyses on somatic cells (BV−) and PGCLCs (BV+) isolated from control PSCs and PSCs with enforced NRF1 expression during their differentiation. We found 2665 genes showed significantly differential expression, with Log_2_ (fold change) below or above 0.58 and *p*‐value <0.05, in BV+ PGCLCs with NRF1 OE versus empty vector control (Figure 5A, Figure S4C, and Table S1). Among these, 1409 genes were upregulated while 1256 genes downregulated (Figure 5B), suggesting NRF1 acts as both a transcription activator and a repressor. The enriched functional categories of differentially expressed genes included several signalling pathways that regulate germ cell development (e.g., *Bmps* and *Wnt*), genes that participate in mitochondrial respiration (e.g., *Cox7a2* and *Atp5e*), and regulators in cell proliferation (e.g., *Jun/Junb*; Figure 5C, Table S1, and Figure S4D). We further confirmed these RNA‐seq results with independent real‐time RT‐PCR analyses (Figure 5D). Notably, the key regulators for PGC formation such as *Blimp1* and *Lin28b* were also upregulated (Figure 5D).

**FIGURE 6:**
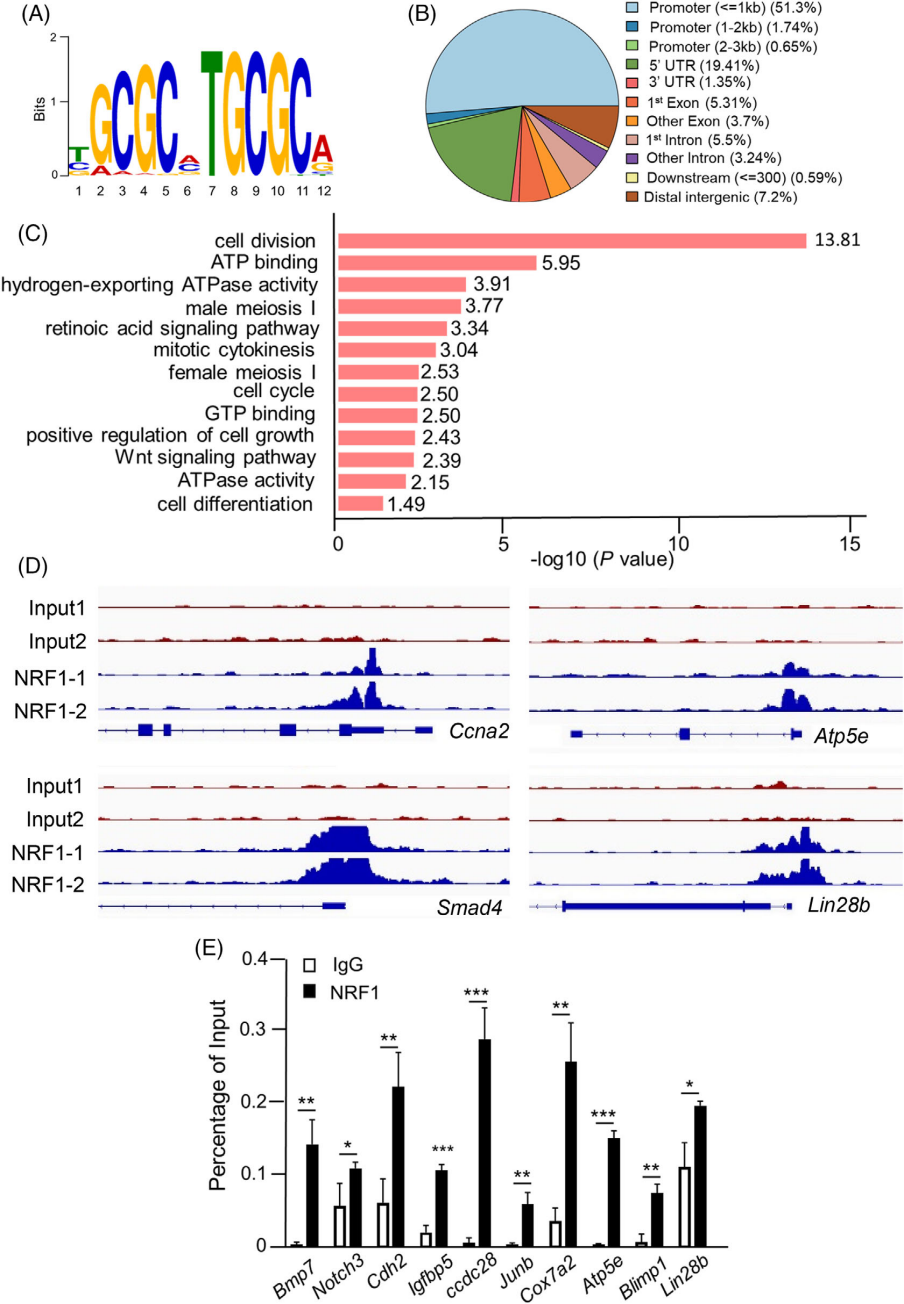
Characterization of NRF1‐mediated transcription network in primordial germ cells (PGCs). (A) NRF1‐binding motif extracted from ChIP‐seq analyses performed on in BV+ PGC‐like cells (PGCLCs). (B) The distribution of NRF1‐binding sites in the genomic regions extracted from ChIP‐seq analyses. (C) Gene ontology analyses of target genes that were bound by NRF1 in BV+ PGCLCs. (D) Representative peaks at the promoters of NRF1 target genes in the genomic regions from ChIP‐seq analyses. (E) Real‐time PCR analyses on immunoprecipitated genomic DNA from BV+ PGCLCs with an NRF1 antibody or with an IgG control. Data were presented as the mean ± SEM from three independent biological replicates. *: *p* < 0.05; **: *p* < 0.01. ***: *p* < 0.001.

To further identify the direct targets of NRF1 in PGC development, we isolated BV+ PGCLCs from wildtype differentiated BVSC PSCs and performed chromatin co‐immunoprecipitation coupled with sequencing (i.e., ChIP‐seq) analyses using NRF1 antibody (Figure S5A,B). In total, we identified 4377 NRF1‐binding genes in PGCLCs with higher than two‐fold enrichment over input control samples (Table S2). A much higher number of genes appeared to be bound by NRF1 than those differentially expressed genes detected by RNA‐seq, suggesting that other NRF1 co‐factors play a critical role in NRF1‐mediated transcriptional regulation. The NRF1‐binding motif detected by ChIP‐seq contained GC‐rich sequences, consistent with the known NRF1 binding sequences (Figure 6A). In addition, in alignment with its role as a transcription factor, more than 51% of NRF1‐binding sequences were located within the gene promoters (Figure 6B). Similar to RNA‐seq results, these NRF1 directly bound genes were highly enriched in functional categories such as WNT signalling, cell cycle and cell differentiation as well as mitochondrial respiration (Figure 6C,D, Table S2, and Figure S5C).

We then cross‐examined RNA‐seq data with ChIP‐seq results and identified 238 upregulated and 222 downregulated genes that were directly bound by NRF1 in BV+ PGCLCs (Table S3). The upregulated NRF1‐bound genes included critical signalling molecules and regulators of PGC specification (e.g., *Bmps*) and germ cell development (e.g., *Notch3*), regulators of cell cycle and proliferation (e.g., *Igfbp5* and *Jun*), and enzymes in mitochondrial respiration and ATP production (e.g., *Cox7a2* and *Atp5e*) (Table S3 and Figures 5D and 6E). Independent real‐time RT‐PCR and ChIP‐PCR analyses further confirmed these findings (Figures 5D and 6E). In summary, we conclude that NRF1 is required for PGC development by regulating their proliferation, survival and mitochondrial metabolism.

## 4 DISCUSSION

PGCs are the initial germline precursors that generate both oocytes and sperm in mammals. PGCs emerge as a small subset of cells during embryonic stages and migrate to genital ridges, where they need to expand before sex determination.^1^, ^2^, ^3^ Albeit the genetic program that segregates PGCs from somatic cells has been extensively studied, it remains elusive how the transcriptional network is regulated to support post‐migrating PGC survival and proliferation. Our current study has uncovered that NRF1, a transcription factor and regulator of mitochondrial biogenesis, plays essential roles in these processes. Our data, in align with published reports,^8^, ^36^ showed that the comparable levels of NRF1 expression between nascent or migrating PGCs and their somatic cell counterparts at the early developmental stages, indicating that NRF1 may not be required for PGC specification. However, we observed that NRF1 expression gradually elevated during embryonic development and was higher in post‐migrating PGCs compared to their surrounding somatic cells. The elevation coincides with the developmental window when global methylation erasure of germ cells occurs,^17^ and given that NRF1 specifically binds to unmethylated promoters,^25^ it is possible that NRF1 exerts its transcriptional regulation mainly in hypomethylated post‐migrating PGCs.

Our study using conditional *Nrf1* knockout further demonstrated that NRF1 plays a critical role in post‐migrating PGC survival and proliferation. *Nrf1* deletion from embryonic germ cells at E9.5 resulted in a significant reduction of Ki67+ proliferating PGCs, along with an increased apoptotic population. Consistent with these findings, our RNA‐seq and ChIP‐seq analyses identified several direct NRF1 target genes that are crucial for cell cycle and cell proliferation. For example, JUN is a basic leucine zipper protein in the AP‐1 complex and an early response activator for cell growth signals.^37^ Our data showed that NRF1 directly binds to the *Jun* gene promoters, and upregulates its transcription, thereby supporting the critical role of NRF1 in promoting PGC proliferation before sex determination.

Although NRF1 might not be required for PGC specification, we observed that increasing NRF1 expression by activating endogenous NRF1 or introducing ectopic NRF1 cDNA enhanced PGC derivation from PSCs. It is plausible that NRF1 specifically promotes nascent PGC proliferation, and thus increases its presence in differentiated PSCs. Alternatively, NRF1 may regulate a transcriptional network that favours PGC specification. Our RNA‐seq results showed that elevated NRF1 expression enhanced several signalling pathways important for PGC development. In particular, previous research indicated that the BMP pathway alone was sufficient to induce pluripotent epiblast cells to differentiate into PGCs.^3^ In addition, BLIMP1 and LIN28b were reported to promote PGC formation from PSCs when upregulated,^8^, ^38^ and our data identified both genes as the direct targets of NRF1. Notably, our ChIP‐seq did not identify *Prdm14* or *Tfap2C*, the other two core transcription factors in PGC specification, as direct targets of NRF1. The enhanced detection of *Prdm14* or *Tfap2C* upon NRF1 expression was likely due to an increased percentage of PGCs in the whole differentiated PSC population. However, both PRDM14 and TFAP2C are involved in other lineage differentiation in addition to germ cell development. When their expression was analysed in the differentiated PSCs, the enhanced PRDM14 and TFAP2C detection from the increased germ cell population may sometimes be masked by the downregulating effects from other lineages. Therefore, the expression of PRDM14 and TFAP2C may not be elevated by NRF1 to the same level as that of BLIMP1 (Figure 4F).

Consistent with the notion that NRF1 is a known regulator of mitochondrial biogenesis, we also found that NRF1 OE led to increased expression of regulators and enzymes involved in mitochondrial respiration. It has been reported that compared to PSCs or surrounding somatic cells, proliferating PGCs have a higher mitochondrial metabolism to meet their energy need. Our data thus implicate that increased mitochondrial activity by NRF1 upregulation favours PGC induction from PSCs, thereby offering a potential application for generating large quantities of PGCLCs from PSCs by optimizing their metabolic profiles. Interestingly, in our previous study, we did not observe significant downregulation of metabolic genes when *Nrf1* was deleted from E15.5 germ cells,^25^ suggesting distinct functional mechanisms by which NRF1 regulates germ cell development at different developmental stages. Taken together, our research provides evidence that NRF1 plays an essential role in regulating post‐migrating PGC survival and proliferation by regulating a broad transcriptional network and mitochondrial metabolism that support PGC programming. These findings shed new light on the functional mechanisms that underlie PGC development and highlight NRF1 as a previously unrecognised regulator of this process.

## AUTHOR CONTRIBUTIONS

Pengxiang Wang, Jun Su, Yilin Xie and Jinhai Zhong performed experiments and analysed data. Junpeng Wang and Wei Chen prepared experimental materials. Yuan Wang supervised the study, designed experiments, interpreted data and wrote the manuscript.

## FUNDING INFORMATION

This work was partially supported by the MSU startup fund and US National Science Foundation (CAREER/IOS 2042908). We thank Dr. Guoliang Xu at the Chinese Academy of Science for providing *Tnap‐Cre* mice, and Dr. Mitinori Saitou at Kyoto University for providing BVSC PSCs.

## CONFLICT OF INTEREST STATEMENT

The authors declare no conflict of interest.

## Supporting information


**Fig. S1. NRF1 is expressed in post‐migrating PGCs.** (**A**) NRF1 expression in PGCs was detected by IHF on E9.5 mouse embryos with NRF1 and STELLA antibodies. Scale Bar: 40 μm. (**B**) Diagram of *Nrf1* conditional knockout in PGCs. (**C**) Representative images of genotyping *Nrf1* conditional knockout mice and PSCs. The mutant allele with floxed Nrf1 locus was generated via homologous recombination, which introduced Frt/LoxP sites surrounding exon 4 and also led to a 72 bp deletion in the intron between exon 4 and 5. Therefore, PCR with primer set 2 (i.e., Loxp‐F and Loxp‐R primers) will yield a 208 bp product from the conditional knockout allele even with a LoxP site, while the wildtype allele will generate a 246 bp product. PCR with this primer set 2 will not be able to amplify any product from the *Nrf1* deleted allele. We also employed the primer set 1 (i.e., Delete‐F and Loxp‐R primers), which can distinguish between *Nrf1* floxed and deleted loci but cannot efficiently amplify a > 8 kb product from the wildtype allele. Combination of primer sets 1 & 2 will allow us to robustly identify wildtype, *Nrf1* floxed, and *Nrf1* deleted alleles.
**Figure S2. NRF1 is required for PGC development.** (**A**) The numbers of OCT4+ or DDX4+ PGCs per gonad section from *Nrf1* deleted embryos and *Nrf1* ^*+/f*^ control embryos during E11.5 to E13.5 were counted based on IHF data. Representative images were shown in Figure 1. Data were presented as the mean ± SEM of target cells calculated from at least 16 gonad sections of three embryos per group. ***: *p* < 0.001. *N.S*.: no significance. (**B**) Examples of genotyping via PCR to determine the sex identity of embryos. PCR was performed on *Rbm31* chromosome loci and produced a 269 bp band from X‐Chr but a 353 bp product from on Y‐Chr. (**C**) IHF was performed on E15.5 male and female gonads from *Nrf1* ^*+/f*^ control embryos and *Nrf1* deleted embryos (*Nrf1* ^*−/f*^ *‐Cre*), with antibodies against NRF1 and DDX4, counterstained with DAPI. Scale bars: 40 μm.
**Figure S3. NRF1 conditional knockout leads to infertility in both male and female mice.** (**A‐B**) Histology study and IHF performed on testis at P0 (**A**) and ovary at P7 (**B**) sections from *Nrf1* ^*−/f*^ *‐Cre* mice vs. their *Nrf1* ^*+/f*^ littermate controls. IHF assays in lower panels were performed with DDX4 and NRF1 antibodies, counterstained with DAPI. No DDX4+ germ cells were detected in either male or female gonads. Scale bars for histology study: 40 μm; for IHF: 60 μm. (**C‐D**) The percentage of OCT4+/Ki67+ proliferating PGCs (**C**) or OCT4+/cleaved Caspase 3+ apoptotic PGCs (**D**) in *Nrf1* conditional knockout embryos and their wildtype controls at E11.5. Representative images were shown in Figure 3D. Data were presented as the mean ± SEM of target cells from 10 gonad sections of three embryos per group. **: *p* < 0.01. ***: *p* < 0.001.
**Figure S4. NRF1 promotes PGCLC formation from PSCs.** (**A**) Representative images of flow cytometry performed on differentiated BVSC PSCs with NRF1 overexpression or with an empty vector control. (**B**) Real‐time RT‐PCR analyses on differentiated BVSC PSCs with NRF1 overexpression or with an empty vector control. Data were presented as mean ± SEM from two independent experiments. (**C**) Heatmap from RNA‐seq analyses on BV− somatic cells and BV+ PGCLCs, with (NRF1 OE) or without (Ctrl) NRF1 overexpression. (**D**) FPKM of representative genes that were upregulated upon NRF1 overexpression (*p20‐Nrf1*) from RNA‐seq analyses on BV+ PGCLCs, compared to empty vector control (*p20*).
**Figure S5. NRF1 direct targets in PGCLCs.** (**A‐B**) BV+ PGCLCs were sorted from differentiated PSCs by flow cytometry (**A**). The ChIP‐PCR analyses (**B**) were done with an NRF1 antibody. All selected genomic regions have been shown to be bound by NRF1 from published studies, using *Rpl30* as a negative control. (**C**) Representative peaks at the genomic region bound by NRF1 from ChIP‐seq analyses.

**Supplemental Table S1.** The list of significantly differentially expressed genes upon NRF1 overexpression in PGCLCs.

**Supplemental Table S2.** The list of genes that are bound by NRF1 in PGCLCs.

**Supplemental Table S3.** The list of NRF1 directly regulated genes in PGCLCs.

**Supplemental Table S4.** The list of oligonucleotides used in this study.

## Data Availability

The ChIP‐seq and RNA‐seq data that support the findings of this study are openly available at GEO repository (https://www.ncbi.nlm.nih.gov/geo/query/acc.cgi?acc=GSE231663; GSE231661 and GSE231662).
