# Supplementary figures and images for: NRF1 promotes primordial germ cell development, proliferation and survival
Source: Cell Prolif. 2023 Aug 4;57(1):e13533. doi: 10.1111/cpr.13533 (PMC10771101; doi:10.1111/cpr.13533)

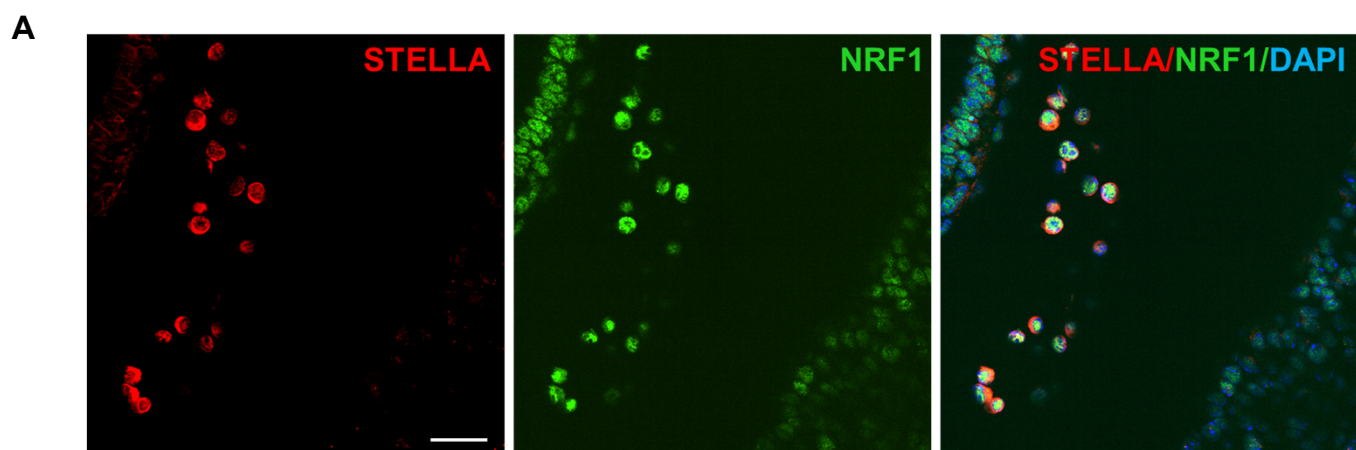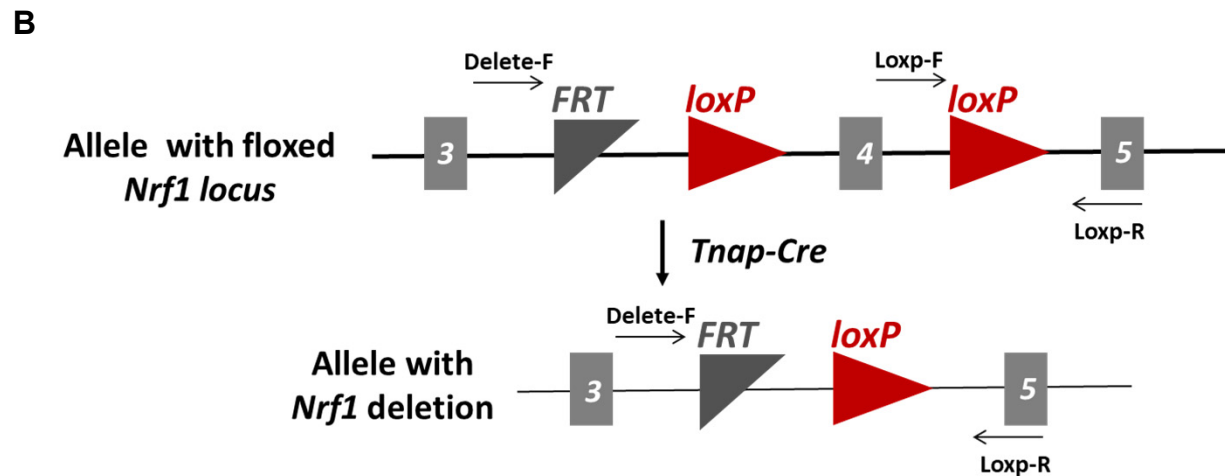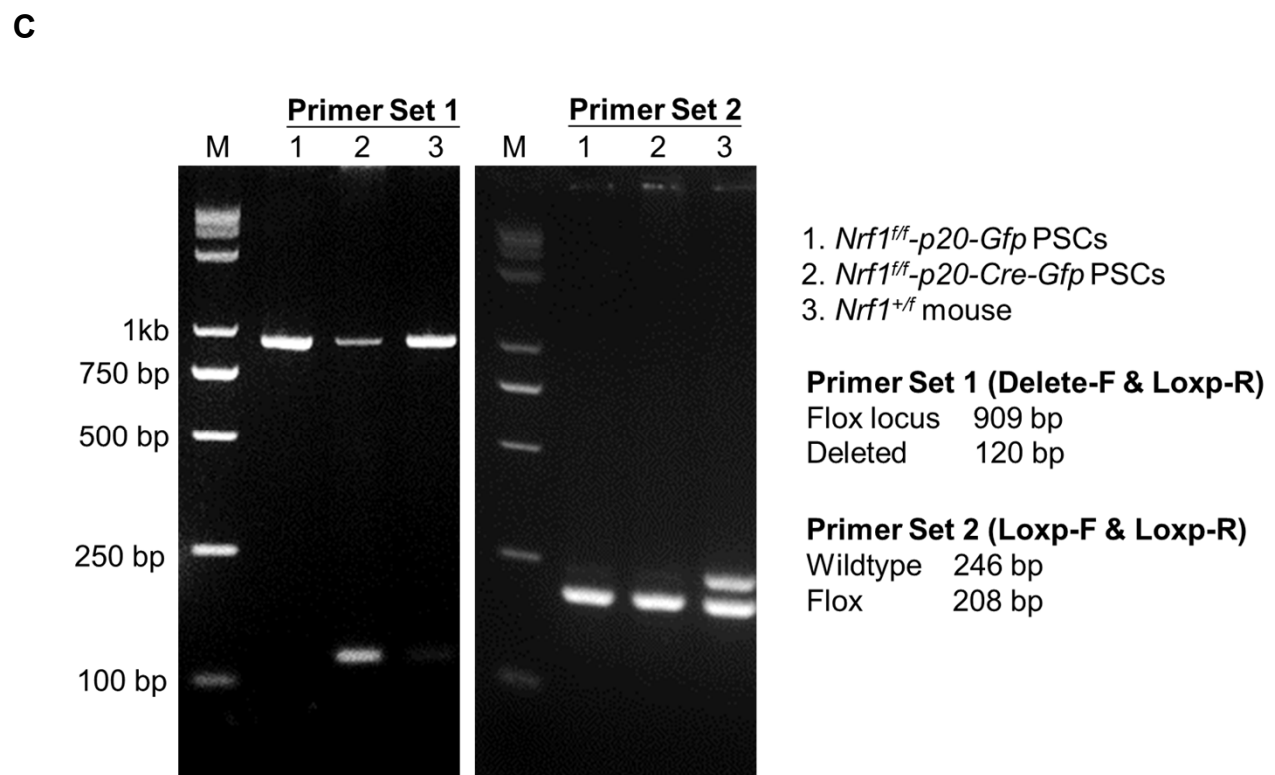

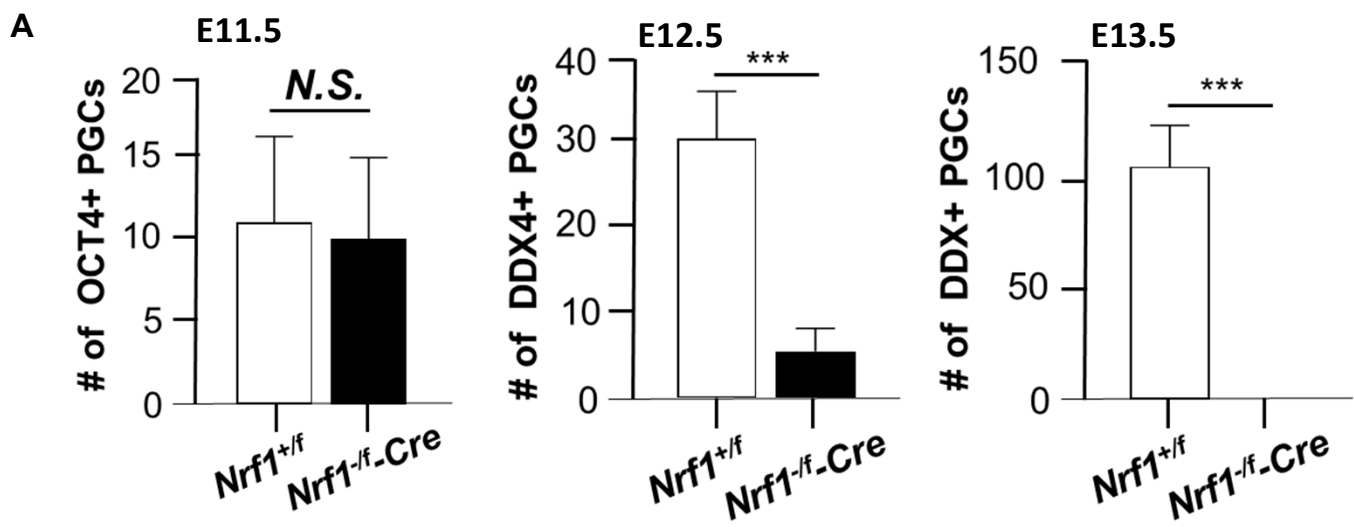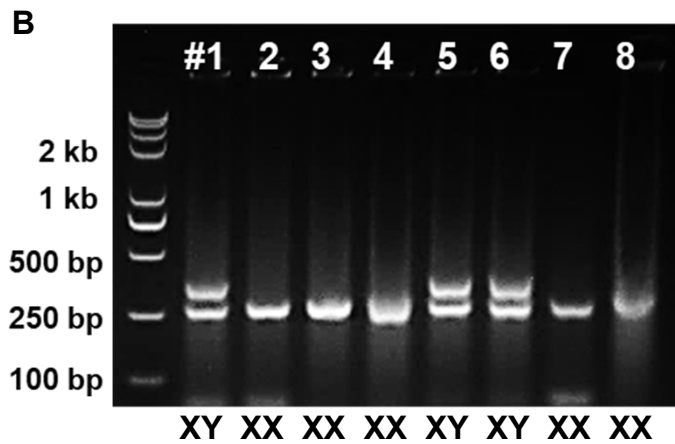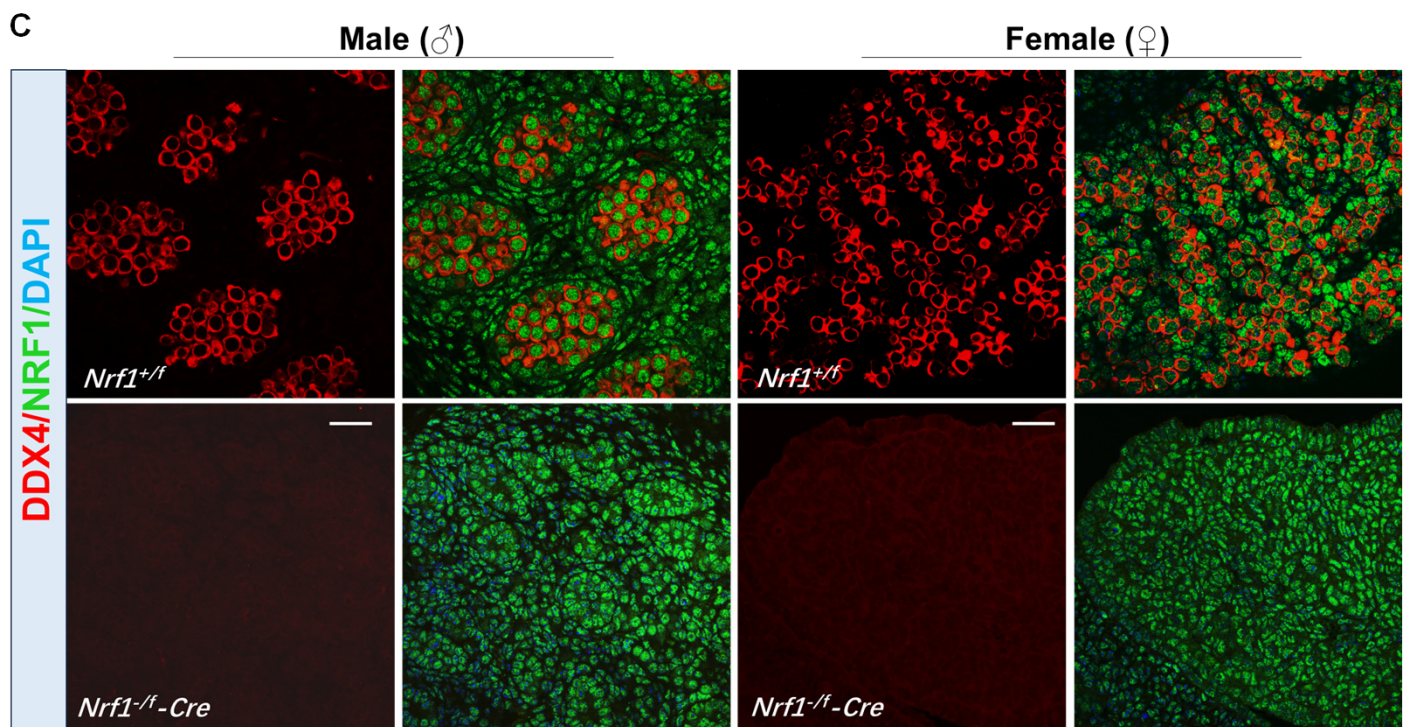

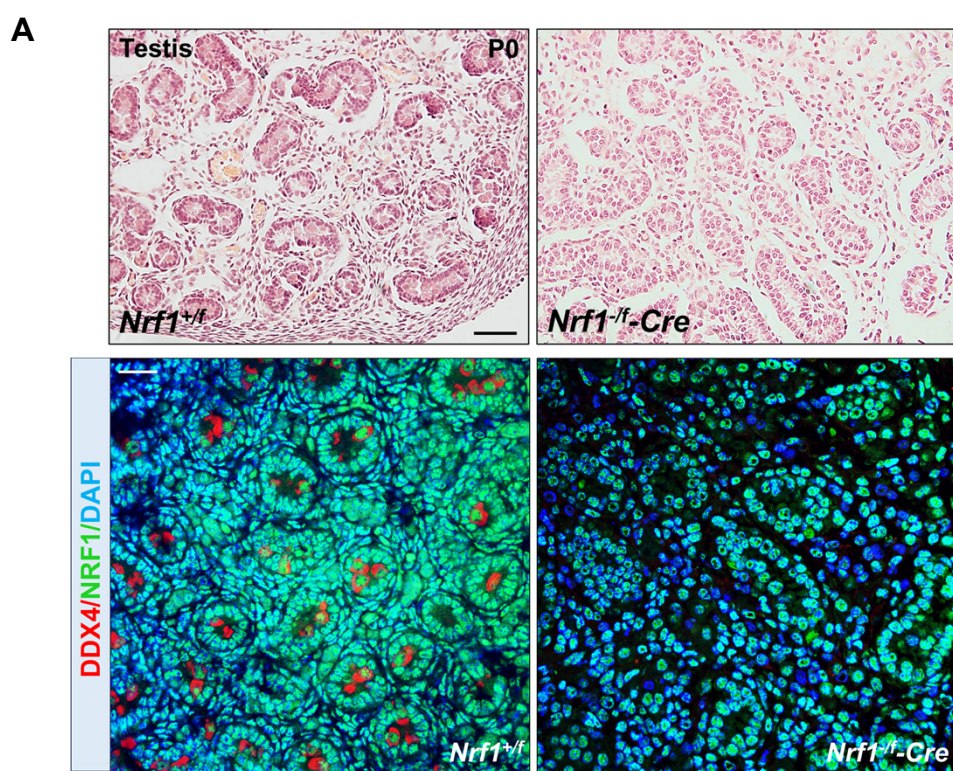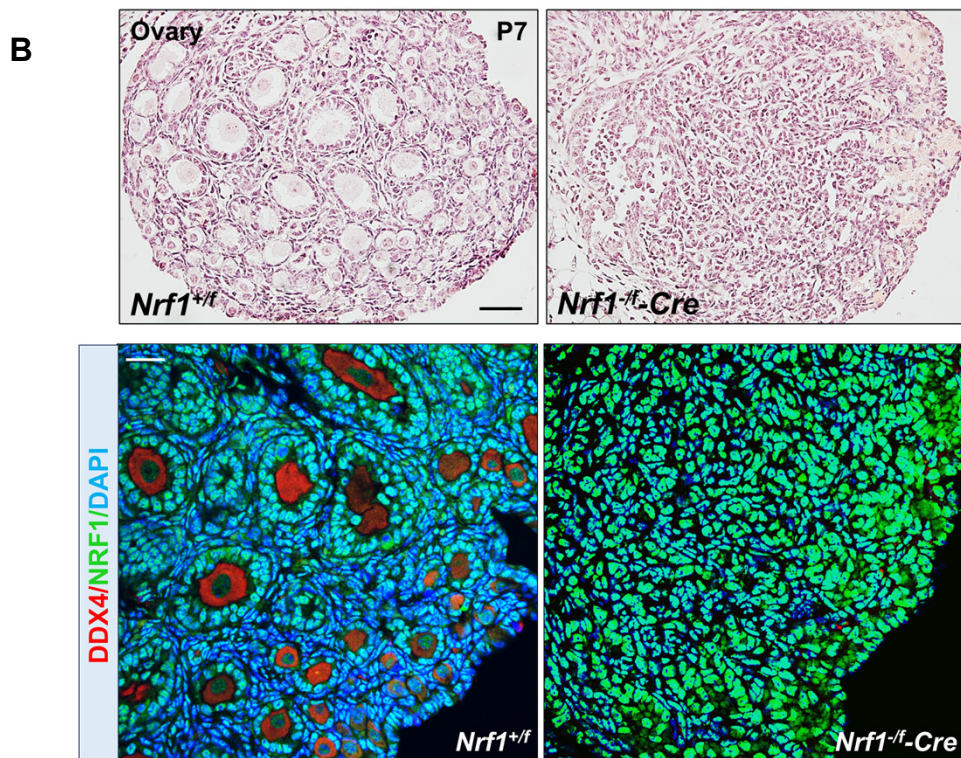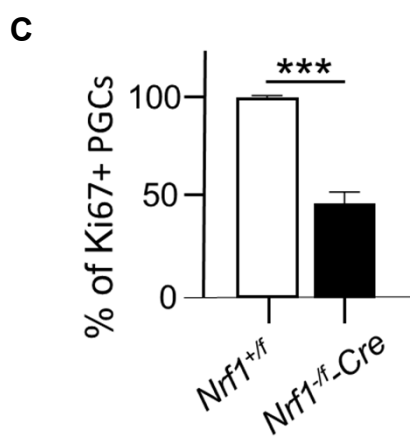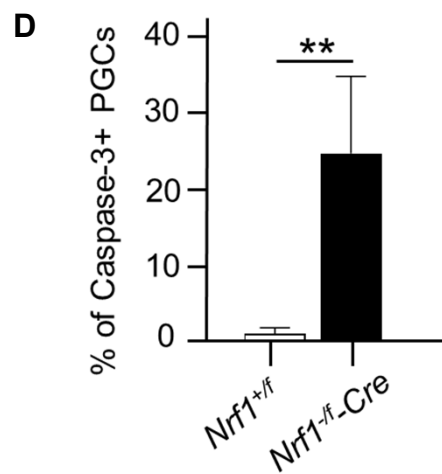

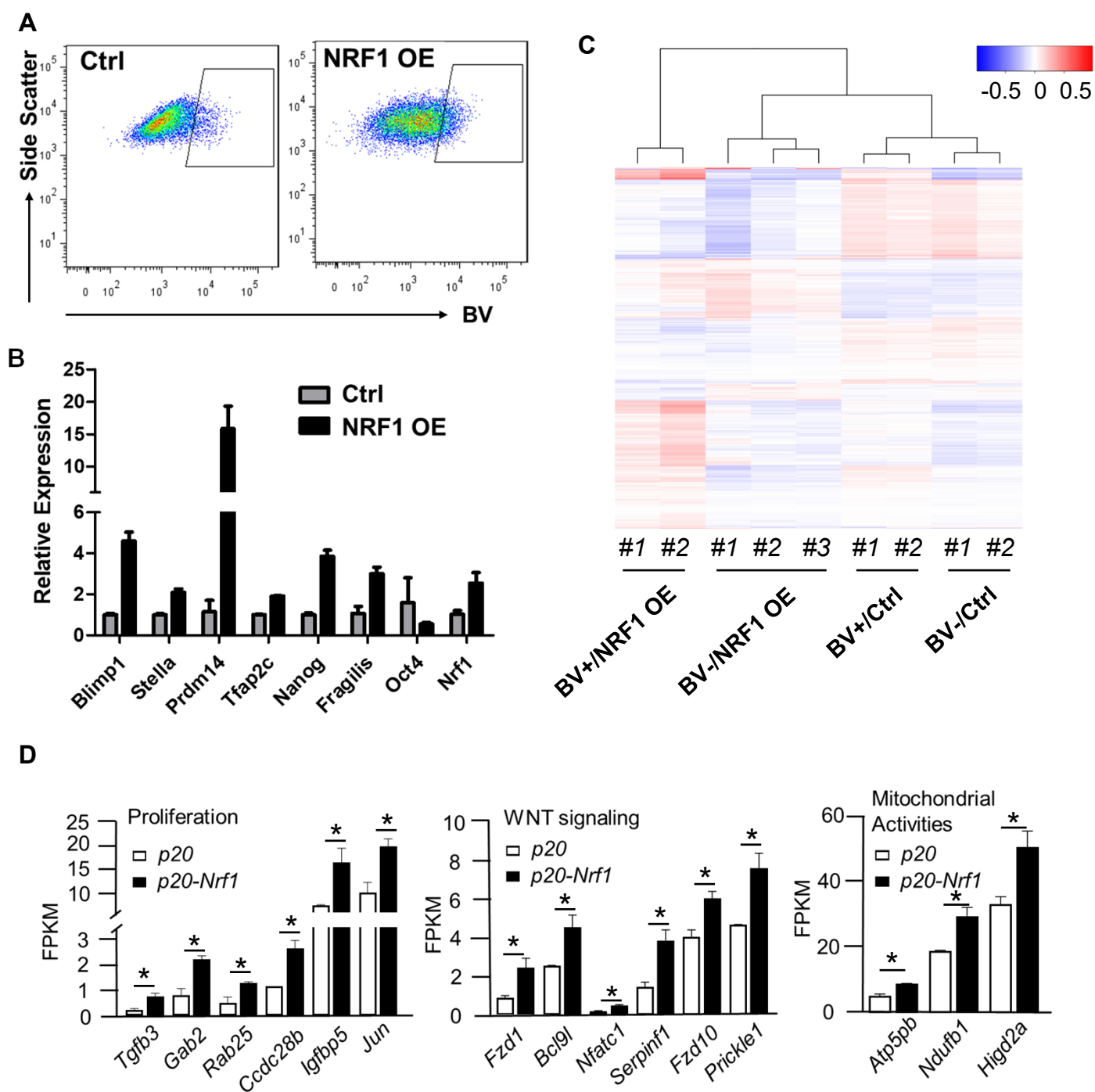

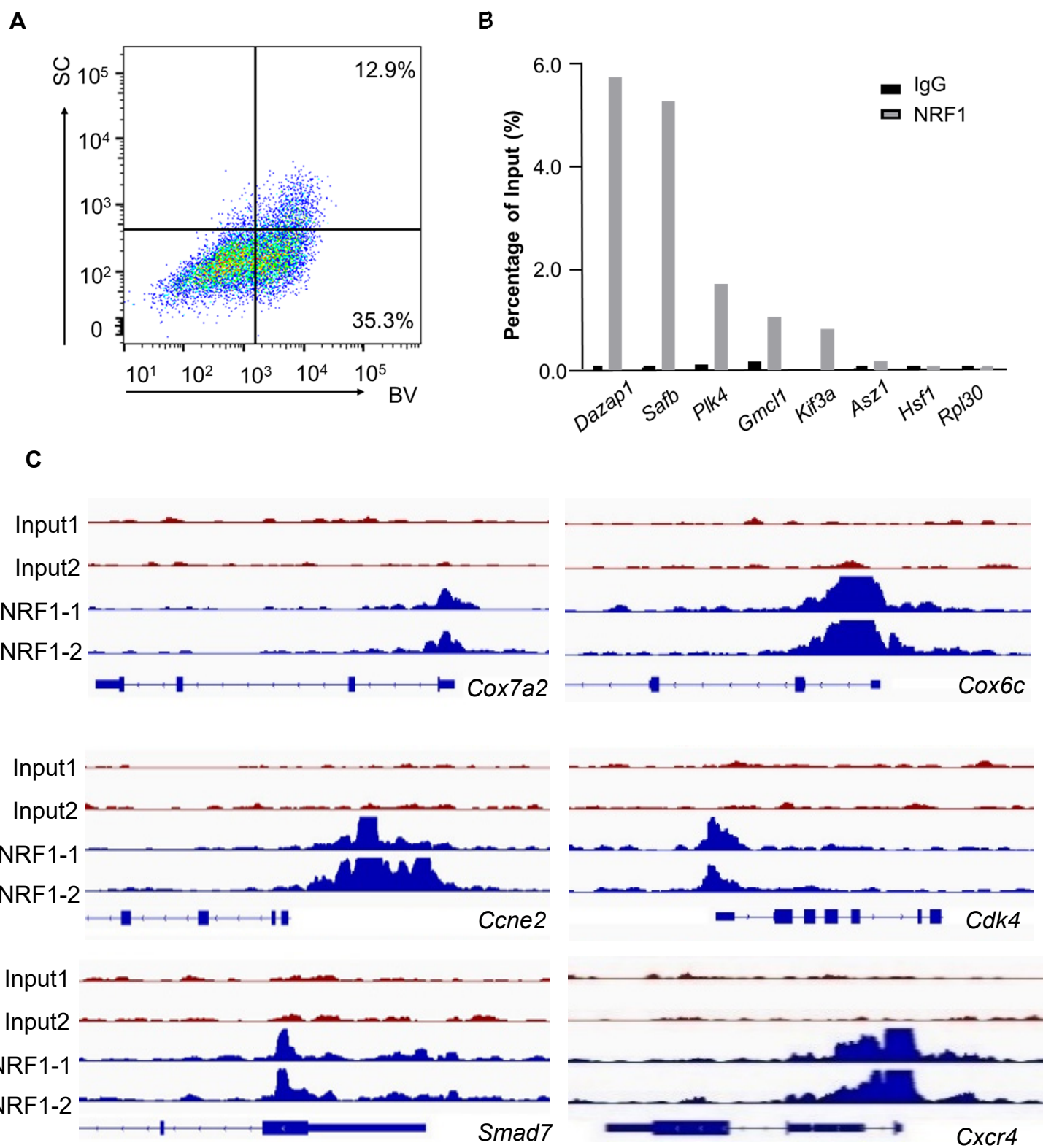

Supplement: Supplementary file 1 — Fig. S1. NRF1 is expressed in post‐migrating PGCs. (A) NRF1 expression in PGCs was detected by IHF on E9.5 mouse embryos with NRF1 and STELLA antibodies. Scale Bar: 40 μm. (B) Diagram of Nrf1 conditional knockout in PGCs. (C) Representative images of genotyping Nrf1 conditional knockout mice and PSCs. The mutant allele with floxed Nrf1 locus was generated via homologous recombination, which introduced Frt/LoxP sites surrounding exon 4 and also led to a 72 bp deletion in the intron between exon 4 and 5. Therefore, PCR with primer set 2 (i.e., Loxp‐F and Loxp‐R primers) will yield a 208 bp product from the conditional knockout allele even with a LoxP site, while the wildtype allele will generate a 246 bp product. PCR with this primer set 2 will not be able to amplify any product from the Nrf1 deleted allele. We also employed the primer set 1 (i.e., Delete‐F and Loxp‐R primers), which can distinguish between Nrf1 floxed and deleted loci but cannot efficiently amplify a > 8 kb product from the wildtype allele. Combination of primer sets 1 & 2 will allow us to robustly identify wildtype, Nrf1 floxed, and Nrf1 deleted alleles. Figure S2. NRF1 is required for PGC development. (A) The numbers of OCT4+ or DDX4+ PGCs per gonad section from Nrf1 deleted embryos and Nrf1 +/f control embryos during E11.5 to E13.5 were counted based on IHF data. Representative images were shown in Figure 1. Data were presented as the mean ± SEM of target cells calculated from at least 16 gonad sections of three embryos per group. ***: p < 0.001. N.S.: no significance. (B) Examples of genotyping via PCR to determine the sex identity of embryos. PCR was performed on Rbm31 chromosome loci and produced a 269 bp band from X‐Chr but a 353 bp product from on Y‐Chr. (C) IHF was performed on E15.5 male and female gonads from Nrf1 +/f control embryos and Nrf1 deleted embryos (Nrf1 −/f ‐Cre), with antibodies against NRF1 and DDX4, counterstained with DAPI. Scale bars: 40 μm. Figure S3. NRF1 conditional knoc [file CPR-57-e13533-s001.pdf]
